# Pigment-Resistant, Portable Corneal Fluorescence Device for Non-Invasive AGEs Monitoring in Diabetes

**DOI:** 10.3390/bios16020087

**Published:** 2026-01-30

**Authors:** Jianming Zhu, Qirui Yang, Jinghui Lu, Ziming Wang, Rizhen Xie, Haoshan Liang, Lihong Xie, Shengjie Zhang, Zhencheng Chen, Baoli Heng

**Affiliations:** 1 School of Life and Environmental Sciences, Guilin University of Electronic Technology, Guilin 541004, China; zhujianming@guet.edu.cn (J.Z.);; 2 Guangxi Academy of Artificial Intelligence, Nanning 530000, China; 3 Infection Management Department, The First Affiliated Hospital of Guangdong Pharmaceutical University, Guangzhou 510080, China; 4 Department of Urology, The First Affiliated Hospital of Jinan University, Guangzhou 510630, China

**Keywords:** AGEs, dietary management, non-invasive detection, blood glucose control

## Abstract

Advanced glycation end products (AGEs) are important biomarkers associated with diabetes and metabolic disorders; yet existing detection methods are invasive and unsuitable for frequent monitoring. This study aimed to develop a non-invasive and portable AGEs detection device, optimize strategies for mitigating pigmentation-related interference, and evaluate its feasibility for metabolic assessment. The proposed system employs a 365 nm ultraviolet LED excitation source, an optical filter assembly integrated into an ergonomic dark chamber, and an eyelid-signal-based algorithm to suppress ambient light and skin pigmentation interference. Simulation experiments were conducted to evaluate the influence of different pigment colors and skin tones on fluorescence measurements. A clinical study was performed in 200 participants, among whom 42 underwent concurrent serum AGEs measurement as the reference standard. Predictive models combining corneal fluorescence signals and body mass index (BMI) were constructed and evaluated. The results indicated that purple and blue pigments introduced greater interference, whereas green and pink pigments had minimal effects. Device-derived AGEs estimates demonstrated good agreement with serum AGEs, with a mean error below 8%. A hybrid model incorporating BMI achieved improved predictive accuracy compared with single-parameter models. Participants with high-AGE dietary habits exhibited elevated fluorescence signals and BMI. These findings suggest that the proposed device enables stable and accurate non-invasive AGEs assessment, with potential utility for metabolic monitoring. Incorporating lifestyle-related parameters may further enhance predictive performance and expand clinical applicability.

## 1. Introduction

The global prevalence of diabetes continues to rise, with an estimated 537 million adults affected in 2021 and projected to reach 783 million by 2045 [1]. The major burden of diabetes arises from chronic hyperglycemia-driven complications, particularly cardiovascular disease and renal failure, which account for most diabetes-related mortality [2]. Early detection and intervention are therefore critical. The American Diabetes Association (ADA) recommends initiating diabetes screening at age 35 and repeating every three years thereafter [3].

HbA1c, the most widely used clinical marker, reflects average glycemia over 2–3 months but is influenced by red blood cell lifespan, anemia, and renal function, and cannot distinguish between stable and fluctuating glycemia [4]. Anemia affects up to one-third of patients with diabetes and is even more prevalent in those with diabetic nephropathy [5,6]. Frequent blood sampling further adds to patient burden [7]. These limitations highlight the need for alternative biomarkers that reliably reflect long-term metabolic status independent of hematologic factors.

Advanced glycation end products (AGEs) are stable compounds formed through nonenzymatic reactions between reducing sugars and proteins, lipids, or nucleic acids [8]. They accumulate under hyperglycemia or through dietary intake of Maillard reaction products [9,10]. Elevated AGEs levels in diabetes are linked to vascular and tissue damage, mediated partly by cross-linking with macromolecules and activation of receptor for AGE (RAGE)-dependent oxidative and inflammatory pathways [11,12,13,14]. Excess AGE accumulation contributes to diabetic complications including nephropathy, cardiovascular disease, and neurodegeneration [15,16,17], making them attractive markers of long-term metabolic imbalance.

Fluorescent AGEs, which constitute a significant proportion of total AGEs, can be detected by fluorescence methods [18,19]. Current approaches—serum, urine, saliva, or skin testing—each face drawbacks: invasiveness, susceptibility to diet and hydration, underdeveloped assays, or interference from aging and pigmentation [20]. Identifying a more stable and interference-resistant site is therefore critical. The cornea, with low pigmentation and high optical uniformity, offers favorable conditions for fluorescence-based detection. Importantly, lens AGEs have been associated with diabetic kidney disease risk independently of HbA1c [21].

As a transparent tissue with low pigment content and excellent optical homogeneity, the cornea provides more accurate conditions for fluorescence detection. Furthermore, studies indicate that lens AGE levels correlate closely with diabetic kidney disease (DKD) risk, independent of HbA1c levels [22]. Consequently, corneal-based AGE detection holds promise as a more reliable, non-invasive alternative for early diabetes screening and risk assessment.

**Figure 1:**
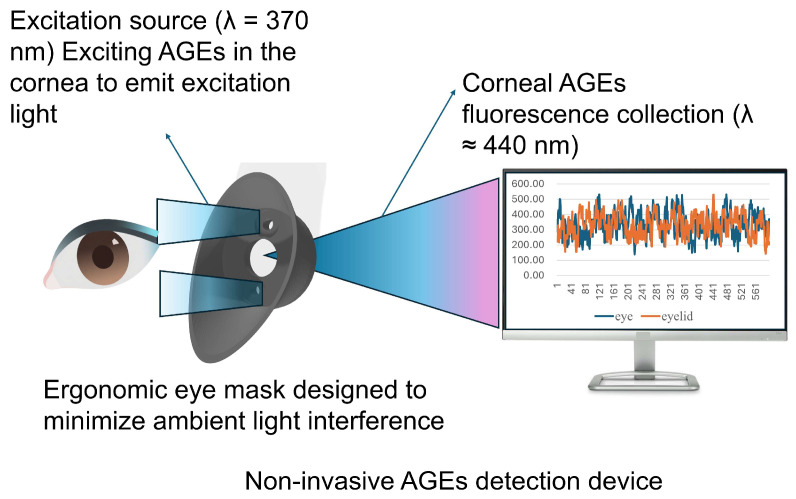
AGE detector assembly.

To address the limitations of existing methods, we developed a portable non-invasive AGE detection device. The system employs a 365 nm ultraviolet LED, optical filters, and a photodiode housed within a 3D-printed ergonomic dark chamber that minimizes ambient light. Based on the international safety standard IEC 60825-1 [23] and the national safety standard GB 7247.1 [24], the 365 nm laser employed in this study is classified as Class 3B at a minimum. Stringent control measures were implemented to limit the single exposure dose to <3.9 mJ/cm^2^, the exposure duration to <10 s, and the beam spot diameter to >7 mm. These parameters ensure that the laser does not cause damage to the subject’s cornea. An eyelid recognition algorithm further reduces interference. We evaluated device performance through pigment interference modeling and preliminary clinical validation to assess stability, robustness, and correlation with diabetes-related indices. Figure 1 shows the detector and an ergonomic 3D eyepiece forming a portable darkroom to minimize light interference, with Bluetooth connectivity enhancing portability.

## 2. Materials and Methods

### 2.1. Pigmentation Interference Modeling

In corneal-based AGE detection, melanin in the iris and choroid may interfere with excitation light and fluorescence signals. Iris color is primarily determined by a combination of melanin (e.g., brown, black, blue, green, gray) [25,26,27] and xanthophyll (e.g., amber), whose density and distribution vary due to individual differences and age-related changes. Therefore, this study established an experimental model simulating melanin interference to provide a foundational basis for subsequent device optimization and data correction. Three types of experiments were designed: qualitative and quantitative fluorescence response experiments, pigment interference resistance experiments, and skin interference validation experiments. Key experimental instruments included a UV-visible spectrophotometer (MAPADA UV-3200 (Shanghai MAPADA Instruments Co., Ltd., Shanghai, China)) and a custom-built light intensity detection device (using VISHAY BPW21R (Vishay Intertechnology, Inc., Malvern, PA, USA) photodiodes identical to the main detector).

For qualitative and quantitative fluorescence experiments, a commercial fluorescent powder (fluorescein sodium-based, analytical grade) was used as the fluorescence source. The powder was dissolved in Ultrapure water (resistivity > 18.2 MΩ × cm) to prepare a series of standard solutions, and Ultrapure water was produced using the Direct-Q system from Merck Millipore. Specifically, 0.01 g of fluorescent powder was dissolved in 100 mL of solvent to obtain an initial concentration of 0.01 g/100 mL, corresponding to 0.01% (*w*/*v*). The concentration was then increased in increments of 0.01 g/100 mL up to 0.05 g/100 mL (0.05% *w*/*v*). All solutions were stored at 4 °C in light-shielded containers to prevent photodegradation prior to measurement.

**Figure 2:**
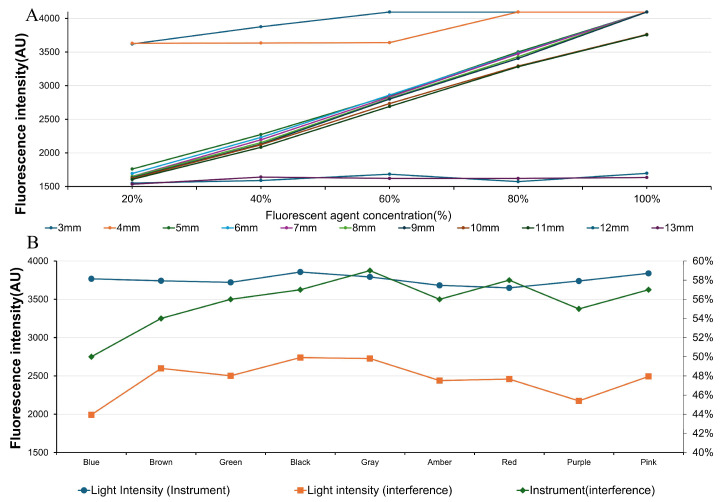
(**A**) illustrates the fluorescence intensity under varying distances and concentrations. (**B**) presents the light intensity data corresponding to different light colors.

During optical detection, the excitation light source and photodetector were arranged in a fixed orthogonal geometry, maintaining a 90° angle between the incident excitation beam and the fluorescence detection axis. This configuration was employed to minimize the influence of specular reflection and scattered excitation light on the detected fluorescence signal. By systematically comparing detection sensitivity under different fluorophore concentrations and source-detector distances, a detection distance of 5 mm and a fluorophore concentration of 0.03 g/100 mL (0.03% *w*/*v*) were selected as the standard parameters for subsequent experiments (Figure 2A).

To analyze pigment interference on detection signals, nine colored pigment solutions (blue, brown, green, black, gray, amber, red, purple, pink) were prepared and added to 60% fluorescent solutions to simulate diverse iris colors. Each color was tested under two device configurations to assess the effects on signal intensity and spectral characteristics (Figure 2B).

**Figure 3:**
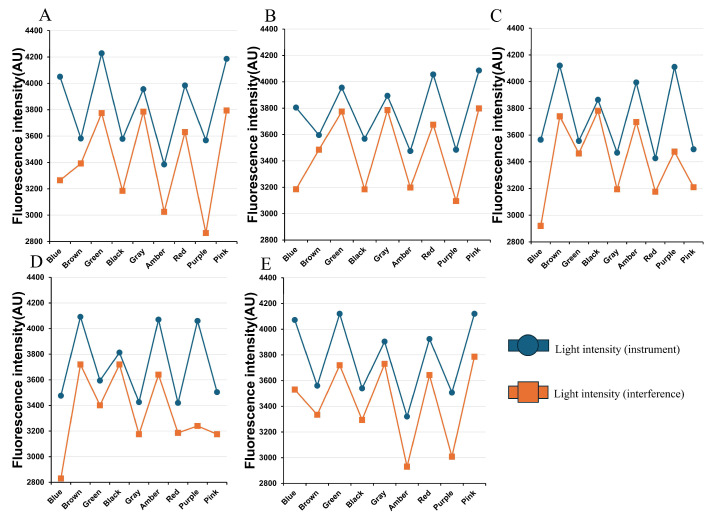
Detection data of different skin tones and colors within the same ethnic group. (**A**) Fluorescence intensity and interference in subjects with the fairest skin tone; (**B**) Fluorescence intensity and interference in subjects with fair skin tone; (**C**) Fluorescence intensity and interference in subjects with moderate skin tone; (**D**) Fluorescence intensity and interference in subjects with dark skin tone; (**E**) Fluorescence intensity and interference in subjects with the darkest skin tone.

Furthermore, to validate potential interference from pigments and skin tones, five volunteers of the same ethnicity but varying skin tones were recruited. Their basic information (age, gender, skin tone, etc.) was recorded, and experiments were conducted in a stable dark environment. Light intensity signals were collected from a fixed 5 cm area on the inner wrist, both before and after staining, with each measurement repeated three times and averaged. Temperature and humidity were maintained constantly to enhance data consistency (Figure 3A–E). Skin tones, which range from light to dark, are designated sequentially as A to E (in the order of increasing darkness).

### 2.2. Clinical Validation Study

To evaluate the accuracy and practicality of this portable AGE detection device in real-world applications, a small-scale preliminary clinical validation study was conducted. The study focused on investigating the correlation between corneal AGE levels and diabetic status, while also assessing the influence of external factors such as lifestyle habits.

#### 2.2.1. Subject Recruitment Criteria

This study enrolled 200 subjects, all adults aged 20–60 years with no history of severe chronic diseases, comprising equal proportions of males and females. Exclusion criteria included severe anemia (Hb < 70 g/L for females; Hb < 80 g/L for males), major ocular diseases, immune system disorders, and pregnancy. All participants signed informed consent forms, and the study was approved by the Ethics Committee of The First Affiliated Hospital of Guangzhou Medical University.

#### 2.2.2. Experimental Procedure

Daily device calibration was performed using NIST-traceable standard fluorescence plates prior to testing. Laboratory conditions were maintained at 23 ± 2 °C/50 ± 5% RH.

Participants also underwent height and weight measurements, with BMI calculated for further analysis. They completed lifestyle questionnaires, primarily including a semi-quantitative food frequency questionnaire (SQFFQ) and dietary preference assessment, with particular focus on the frequency of consuming foods prepared via high-temperature cooking methods (e.g., grilling, frying).

This study recruited 200 participants of the same ethnicity. All subjects first underwent portable non-invasive AGE detection device measurements of corneal and eyelid fluorescence signals in both eyes, with three repeated measurements averaged. Among them, 42 subjects underwent additional blood sampling: 5 mL of fasting venous blood was collected and analyzed via ELISA to determine serum AGE levels as the gold standard reference. Seventeen participants had kidney-related diseases such as diabetic nephropathy and were undergoing treatment. Some of the Detection results and error values are shown in.

#### 2.2.3. Data Processing and Model Construction

**Figure 4:**
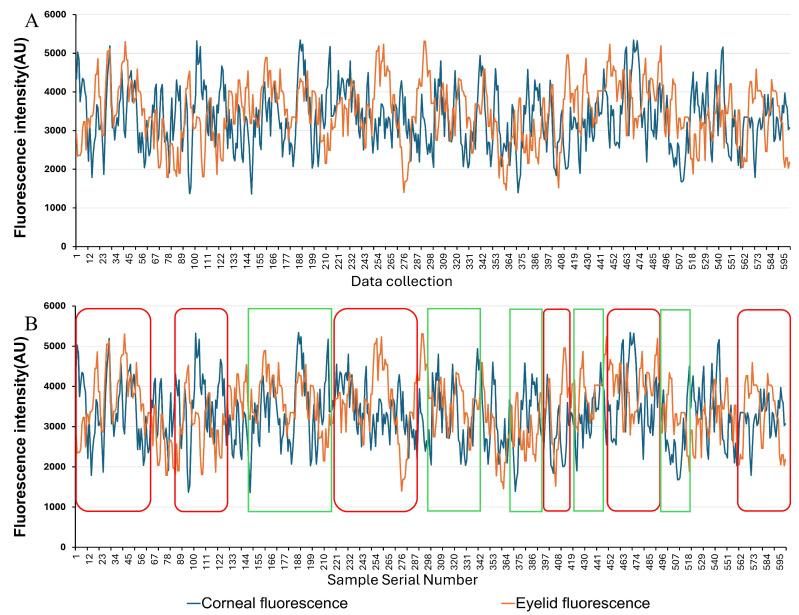
Clinical data processing and detection analysis of the cornea and eyelids. (**A**) Unprocessed data. (**B**) Processed data. The corneal fluorescence data within the green box shows an opposite trend to the eyelid fluorescence data, indicating the presence of interference. The data within the red box exhibits a consistent trend and is regarded as valid data.

Due to its inherently low emission intensity, corneal fluorescence signals are susceptible to interference from ambient light and reflections. To enhance signal purity, this study introduced eyelid fluorescence signals as a correction factor, employing feature signal recognition and data processing methods to eliminate interference components. Figure 4A shows raw, unprocessed data obtained from the detector test. The horizontal coordinate data represents the sample serial number. Figure 4B shows processed data. The eyelid data has been separated from the corneal data. The corneal fluorescence data within the green box exhibits a trend opposite that of the eyelid fluorescence data, indicating interference from ambient light or reflected light. In contrast, the data within the red box shows a consistent overall trend and is considered valid data, retained for subsequent preprocessing (Figure 4).

Based on denoised corneal data, three prediction models were constructed: multiple linear regression (MLR), support vector regression (SVR), and a weighted hybrid model combining both, as shown in Equation (1). Using serum AGE measurements as the reference standard, models were trained and validated with corneal fluorescence signals and BMI as input features.

(1) y=0.7×MLR(x)+0.3×SVR(x)

(2) y=0.7×MLR(x)+0.3×SVR(x)

Multiple linear regression (MLR) and support vector regression (SVR) were used to estimate AGEs based on extracted optical features. The MLR model was trained using ordinary least squares. Multicollinearity was mitigated through feature selection rather than explicit regularization.

The SVR model employed a radial basis function (RBF) kernel, with hyperparameters optimized via grid search on the training set (C = 10, ϒ = 0.1, ε = 0.01). Feature selection was performed using Pearson correlation analysis combined with variance thresholding, excluding features with low variance or weak correlation with serum AGEs (|r| < 0.3).

A hybrid prediction model was constructed by linearly combining the outputs of the MLR and SVR models. The weighting coefficients (0.7 for SVR and 0.3 for MLR) were determined based on validation performance, as the SVR model demonstrated superior nonlinear fitting and lower prediction error. This ensemble strategy enhanced robustness by leveraging the complementary strengths of linear and nonlinear regression.

**Figure 5:**
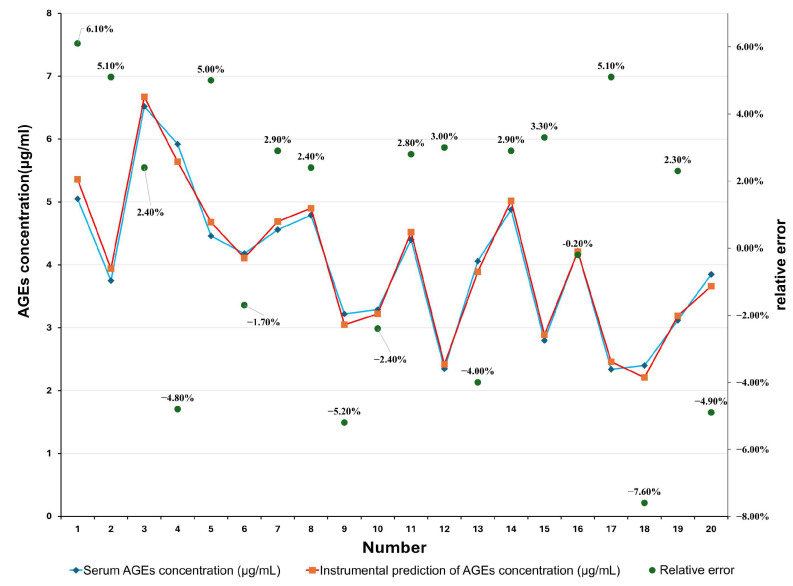
Data processing and analysis.

Comparative results indicate that while age alone exhibits correlation in certain ranges, its predictive stability is constrained by individual metabolic variations. Incorporating BMI enables the model to better reflect lifestyle and metabolic differences, enhancing predictive accuracy and generalization capability. Among the three models, the mixed model demonstrated the most stable performance and optimal predictive capability. Compared to other approaches, incorporating BMI can more effectively captures the influence of individualized factors like lifestyle habits on AGEs accumulation, exhibiting superior predictive ability. Clinical data and instrument test data are shown in Table 1 and Figure 5. The following conclusions can be drawn from the data presented in Table 1: Pearson correlation analysis demonstrated a strong positive correlation between corneal fluorescence-derived AGE values and serum AGE measurements (r = 0.991, *p* < 0.001). Bland-Al-man analysis showed a mean bias of −0.029 with 95% limits of agreement ranging from −0.347 to 0.290, indicating good agreement between corneal fluorescence measurements and serum AGE reference values.

## 3. Results

### 3.1. Experimental Conclusion

#### 3.1.1. Pigment Interference Experiments

Fluorescence measurements demonstrated differential interference across pigment solutions. Pigments corresponding to purple and blue light exhibited the most pronounced attenuation of fluorescence intensity, primarily due to the spectral overlap between these pigments and the excitation light, blue, and purple pigments exhibit higher absorption coefficients and stronger scattering within this spectral range, leading to attenuation of excitation light and partial masking of emitted fluorescence signals., whereas green and pink pigments had comparatively weaker effects. Variation in skin tone slightly modulated the degree of signal interference; however, differences remained within a stable range. Signal repeatability was preserved across pigment and skin-tone combinations, indicating that the system maintained consistent performance despite external optical interference. These findings suggest that, under stable environmental conditions, the influence of the iris or pupil color on AGE fluorescence detection is minimal.

#### 3.1.2. Clinical Validation

A total of 200 subjects were enrolled, of whom 42 underwent concurrent serum AGE testing. Corneal fluorescence values showed good agreement with ELISA-based serum AGE concentrations, with an average absolute error of less than 8%. Accuracy was particularly consistent among individuals with elevated AGE levels, supporting the sensitivity of the device in detecting higher concentrations.

When eyelid reflection artifacts were excluded, predictive modeling that integrated corneal fluorescence intensity with BMI demonstrated improved estimation of AGE content. The hybrid model achieved superior fit across datasets compared with fluorescence-only models, confirming its robustness and adaptability after physiological calibration.

The portable, non-invasive AGE detection device developed in this study demonstrates robust performance in controlling pigment interference and maintaining clinical applicability. By integrating eyelid signal-assisted calibration and optimizing the device structure, the system effectively minimizes reflection artifacts arising from ambient light and variations in pigmentation. In clinical validation, corneal fluorescence intensity measured by the device correlated well with serum AGE concentrations, with mean absolute errors of less than 8%, and predictive accuracy was particularly high in individuals exhibiting elevated AGEs accumulation.

## 4. Discussion

Analysis of the predictive model revealed a systematic bias: at higher AGE concentrations, predicted values tended to slightly overestimate serum levels, whereas at lower concentrations, a minor underestimation was observed; several factors may contribute to this phenomenon. At higher fluorescence intensities, partial signal saturation or nonlinear response of the photodetector may occur, leading to compression of the dynamic range. Conversely, at low AGE levels, background noise and baseline fluorescence may exert a proportionally greater influence on the measured signal.

From an algorithmic perspective, the use of linear or mild nonlinear regression models may also contribute to regression-to-the-mean effects. Future work will explore nonlinear calibration strategies, detector linearity correction, and dynamic range normalization to reduce systematic bias across the full AGE concentration range.

Dietary habits were found to significantly influence AGE levels; participants with a preference for high-AGE foods, such as barbecued or fried items, exhibited both higher fluorescence signals and BMI, indicating a close association between lifestyle factors and AGE accumulation. In the future, further consideration can be given to the impact of dietary content on fluorescence data.

This study represents a first attempt to incorporate eyelid fluorescence data into a non-invasive detection system to eliminate invalid signals, while including BMI as an auxiliary parameter reflecting individual metabolic status. This approach enhanced model adaptability and generalization across subjects, enabling more reliable, quantitative estimation of AGE content. The device offers notable advantages, including non-invasiveness, rapid measurement, portability, and ease of use, supporting its potential for broader clinical application.

Limitations include a relatively small sample size and a population restricted to a single ethnicity, highlighting the need for validation in larger and more diverse cohorts. First, the number of subjects undergoing serum AGE reference testing was relatively limited, which may restrict the statistical power for subgroup analyses. Second, the clinical chore was recruited from a single ethnic population, potentially limiting the generalizability of the findings to populations with different iris color distributions, skin pigmentation characteristics, and ocular anatomical features. Future studies with larger, multi-ethnic cohorts and stratified analyses based on pigmentation characteristics are warranted to further validate the robustness and clinical applicability of the proposed corneal fluorescence detection approach.

Although subjects with major ocular diseases were excluded, common non-severe ocular conditions may still influence corneal fluorescence measurements. Early stage cataracts can increase intraocular light scattering, potentially affecting excitation delivery and fluorescence collection. Long-term contact lens use may alter corneal hydration and epithelial thickness, which could modify optical transmission properties. In addition, dry eye syndrome may affect tear film stability, leading to variability in surface reflectance.

While these factors were not explicitly controlled in the present study, their potential influence should be considered in future investigations. Incorporating ocular surface assessments and stratified analyses may further improve measurement robustness in real-world clinical settings.

Future research will expand the population base and geographical coverage while integrating additional lifestyle parameters, such as dietary patterns and physical activity, into the predictive model. Such refinements could provide more direct indicators of personalized metabolic status than BMI alone, thereby improving the accuracy and clinical utility of non-invasive AGE detection for early diabetes risk assessment and disease monitoring.

## Figures and Tables

**Table 1:** Comparison of clinical data and instrumental testing data.

Serial Number	Serum AGE Concentration Testing (μg/mL)	Instrument Predicts AGE Concentration (μg/mL)	Relative Error (%)
1	5.05	5.36	6.10
2	3.75	3.94	5.10
3	6.52	6.67	2.40
4	5.92	5.64	−4.80
5	4.46	4.68	5.00
6	4.18	4.11	−1.70
7	4.56	4.69	2.90
8	4.79	4.90	2.40
9	3.22	3.05	−5.20
10	3.29	3.22	−2.40
11	4.40	4.52	2.80
12	2.35	2.42	3.00
13	4.06	3.89	−4.00
14	4.88	5.02	2.90
15	2.80	2.89	3.30
16	4.22	4.21	−0.20
17	2.34	2.46	5.10
18	2.40	2.21	−7.60
19	3.12	3.19	2.30
20	3.85	3.66	−4.90

## Data Availability

The datasets generated and analyzed in this study are not publicly available because they contain confidential human participant data and public sharing was not included in the informed consent. To protect participant privacy and comply with ethical and legal requirements, access to the data is restricted. The data may be provided upon reasonable request, subject to ethical approval and data protection regulations.

## Abbreviations

The following abbreviations are used in this manuscript:

AGEs	Advanced glycation end products
